# Understanding Fatigue in Sjögren’s Syndrome: Outcome Measures, Biomarkers and Possible Interventions

**DOI:** 10.3389/fimmu.2021.703079

**Published:** 2021-06-25

**Authors:** Elisabeth Mæland, Samira T. Miyamoto, Daniel Hammenfors, Valeria Valim, Malin V. Jonsson

**Affiliations:** ^1^ Department of Rheumatology, Haukeland University Hospital, Bergen, Norway; ^2^ Department of Integrated Education in Health, Federal University of Espírito Santo, Vitoria, Brazil; ^3^ Broegelmann Research Laboratory, Department of Clinical Science, University of Bergen, Bergen, Norway; ^4^ Department of Rheumatology, University Hospital of Federal University of Espírito Santo (HUCAM-UFES/EBSERH), Vitoria, Brazil; ^5^ Department of Clinical Dentistry, Section for Oral and Maxillofacial Radiology, University of Bergen, Bergen, Norway; ^6^ Research Department, Oral Health Centre of Expertise in Western Norway, Bergen, Norway

**Keywords:** Sjögren’s sydrome, fatigue, outcome measure, cytokines, biomarker (BM), intervention

## Abstract

Sjögren’s syndrome (SS) is an autoimmune disease affecting the salivary and lacrimal glands. Symptoms range from dryness to severe extra-glandular disease involving manifestations in the skin, lungs, nervous system, and kidney. Fatigue occurs in 70% of patients, characterizing primary SS (pSS) and significantly impacting the patient’s quality of life. There are some generic and specific instruments used to measure fatigue in SS. The mechanisms involved with fatigue in SS are still poorly understood, but it appears fatigue signaling pathways are more associated with cell protection and defense than with pro-inflammatory pathways. There are no established pharmacological treatment options for fatigue in pSS. So far, exercise and neuromodulation techniques have shown positive effects on fatigue in pSS. This study briefly reviews fatigue in pSS, with special attention to outcome measures, biomarkers, and possible treatment options.

## Introduction

Sjögren’s syndrome (SS) comprises a wide range of symptoms and disease severity. The disease predominately affects middle-aged women with a 9:1 female:male ratio. The prevalence ranges from 0.04 to 0.17 (1). Hallmark complaints are dryness and fatigue (2, 3). Systemic manifestation can affect 50-70% of patients. Some patients have increased risk of lymphoma, usually associated with cryoglobulinemia, systemic manifestations, purpura, parotid enlargement, and pronounced salivary gland inflammation (high focus score) (4).

Fatigue is a complex and multi-faceted phenomenon, defined as a feeling of physical tiredness and lack of energy (5). The prevalence of fatigue in primary Sjögren’s syndrome (pSS) is approximately 65-70%, and often reported as the most debilitating symptom (6). Next to pain and dryness, fatigue was one of the most common complaints that the patients would like to have improved (7).

A clinical and laboratory investigation of 141 patients found that fatigue in pSS did not change significantly over time (8). Thirty-five percent of patients experienced a clinically significant increase in fatigue severity, but fatigue measures did not change on group level. Multivariate models were not able to reveal clinical or laboratory predictors of fatigue change over time.

In line with these findings, a qualitative study encompassing nine patients with pSS and fatigue, describes fatigue as an ever-present, fluctuating, and uncontrollable lack of energy, beyond the individuals own control. Fatigue in pSS clearly differs from ordinary tiredness and may have a considerable impact on a patient’s life (3). Functional impairment has also been associated with physical fatigue in pSS, with patients experiencing significant functional disability compared to age-matched healthy controls. The impaired function was associated with reduced quality of life (QoL) and symptoms such as pain and depression as well as disease activity, illustrating the need for management of several disease aspects (9).

Compared to healthy controls (n=168), QoL was considerably impaired in patients with pSS (n=185), with pain, fatigue, disease activity, impaired swallowing, and anxiety/depression remaining as main predictors of QoL in the linear regression model (10). Another study found fatigue and pain to be the main predictors of poor QoL in patients with pSS regardless of disease activity, age, schooling, marital status, work disability and fibromyalgia (11). Though such complications are not life-threatening, the chronicity can lead to heavy debility and reduced quality of life, affecting not only daily activities and social life, but also career and finances (12).

The underlying mechanisms of fatigue are not completely understood, but increasing evidence suggests a multifaceted picture. An interesting idea is the sickness behavior phenomenon observed during infection and inflammation. Fatigue constitutes a substantial part of this behavior, to increase survival during temporary infections or harm. In chronic diseases fatigue becomes chronic without a meaningful purpose (13, 14). Based on observations from rheumatoid arthritis (RA), where some patients fatigue improve markedly following treatment with biological agents, whereas others continue to suffer from fatigue despite clinical remission, genetic and epigenetic variations have been suggested (13).

The overall aim was to briefly review fatigue in SS with special attention to outcome measures, biomarkers and current treatment options.

## Instruments for Measuring Fatigue In PSS

The Profile of Fatigue and Discomfort-Sicca Symptoms Inventory (PROFAD-SSI), whose fatigue component (Profile of Fatigue – Prof-F) measures the somatic (ProF-S) and mental (ProF-M) fatigue, and the EULAR Sjögren’s Syndrome Patients Reported Index (ESSPRI), which uses 0-10 numerical scales for the assessment of dryness, fatigue, and musculoskeletal pain domain, are two SS-specific instruments for measuring fatigue. Future studies may prefer to use one of these two instruments or both. The development of objective measures of fatigue depends on the elucidation of the pathophysiological mechanism of fatigue in pSS, which is still unclear.

The 10-cm fatigue visual analogue scale (VAS) provides a global fatigue score where a higher score represents greater severity or intensity of fatigue. VAS is often used in pSS research, probably due to its simplicity. However, a multidimensional assessment may provide a more complete picture, improving understanding of the clinical associations of fatigue and potential treatment. Studies on pSS have also used many non-disease specific multi-item questionnaires (15, 16) which would be most recommended when one of the study objectives is to compare fatigue across diseases. Commonly used instruments are summarized in **Table 1**.

**Table 1:** Current fatigue measures used for Sjögren’s syndrome.

Acronym	Name and description	Applicability: strengths/limitations
**Specific instruments in pSS**			
PROFAD-SSI	Profile of Fatigue and Discomfort – Sicca Symptoms Inventory (Short-form) PROFAD: nine items split into four domains – arthralgia, vascular, somatic fatigue (ProF-S), mental fatigue (ProF-M) SSI: 10 items split into four domains – ocular dryness, oral dryness, vaginal dryness, and cutaneous dryness In both the final score is the sum of its four domains and varies from 0 to 28.	Profile of fatigue (Prof-F) of the PROFAD has the advantage of distinguishing fatigue in physical and mental types in pSS. Even if somatic fatigue is the predominant domain, all domains have the same weight on the PROFAD final score.
ESSPRI	EULAR Sjögren’s Syndrome Patients Reported Index 0 to 10 numerical scales for the assessment of each of the three domains: dryness, fatigue and musculoskeletal pain. The mean of the scores of the 3 domains represents the final score. ESSPRI ≥ 5: unsatisfactory symptom state. Clinically meaningful improvement: at least one point or 15%.	Recommended by EULAR, quick and simple to administer and score. Does not capture the multidimensional nature of fatigue.
**Non-disease specific single-item instrument**			
VAS	Visual Analogue Scale 0-100 cm or 0-10 cm with a higher score representing a greater severity or intensity of fatigue.	One of the most frequently used tools to measure fatigue. Quick and simple to administer and score, and minimal in terms of respondent burden. Does not capture the multidimensional nature of fatigue.
**Non-disease specific multi-item questionnaires**			
FACIT-fatigue	Functional Assessment of Cancer Therapy Scale – fatigue 13 items covering physical fatigue, functional fatigue, emotional fatigue, and social consequences of fatigue. Final scores produce only a global score ranging from 0–52, with higher scores reflecting less fatigue.	FACIT-Fatigue is used across many rheumatologic conditions, covering a range of fatigue concepts in a simple language. It provides only a global fatigue score.
FSS	Fatigue Severity Scale 9 items to produce a global score ranging from 1-7 with higher scores reflecting greater fatigue. The FSS covers physical, social, or cognitive effects of fatigue.	It provides only a global fatigue score. Recommended fatigue scale for systemic lupus erythematosus more than pSS.
MFI	Multidimensional Fatigue Inventory 20 items, yielding 5 subscales of 4 items each (general fatigue, physical fatigue, reduced activity, reduced motivation, and mental fatigue). Scores of each subscale range from 4–20 with higher scores reflecting greater severity.	It provides 5 subscales of fatigue. The wording of some items may be interpreted as relating to disability or disease activity.
FIS	Fatigue Impact Scale 40 questions covering the effect of fatigue on three domains of daily life: cognitive functioning, physical functioning, and psychosocial functioning. There is an overall score with a potential maximum of 160. A score of ≥ 40 indicates excessive symptomatic fatigue and ≥ 80 indicates severe, symptomatic fatigue. Subscale scores can also be calculated.	It provides the effect of fatigue on three domains of daily life. Few studies in pSS.
CFS	Chalder Fatigue Scale 11 items to produce a global score (0-33 or 0-11) and 2 domains of physical (0-21) and mental fatigue (0-12) with higher scores reflecting greater fatigue.	It provides physical and mental fatigue domains. It does not always differentiate between rheumatology patients and controls.
SF-36 VT	Medical Outcomes Study Short-Form (Domain: Vitality) 4 items in the SF-36 VT (2 on energy and 2 on fatigue). Scores range from 0–100 with higher scores representing less fatigue.	The SF-36 VT has been used across many rheumatologic conditions and in many studies. It provides only a global fatigue score and there are concerns over concepts of fatigue *vs*. energy.

## Biomarkers to Understand Fatigue

Interestingly, fatigue is not associated with systemic disease activity (11, 17). In agreement with this clinical observation, although pSS is an autoimmune inflammatory disease, there is a paradoxical inverse correlation of fatigue with pro-inflammatory cytokines (18, 19).

A panel of 14 cytokines that were elevated in patients with pSS were compared to controls without fatigue. However, when analyzing only a subgroup of pSS with fatigue, the fatigue scores were inversely proportional to four pro-inflammatory cytokines, interferon-γ inducible protein 10 (IP-10), tumor necrosis factor α (TNF-α), lymphotoxin α (LT-α), and interferon γ (IFN-γ) (18). Results were confirmed in a larger sample, showing that IP-10, TNF-α, interferon- α (IFN-α), IFN-γ, and LT-α were significantly higher in patients with pSS (n=120) compared to non-fatigued controls (n=30), with TNF-α and LT-α inversely related to patient-reported levels of fatigue (19).

On the other hand, pain, depression, and daytime sleepiness scores were closely associated with both physical and mental fatigue in pSS, with effects observed even after adjustment for comorbidities associated with fatigue or medications associated with drowsiness (20).

Taking cytokine levels, disease-specific and clinical parameters in a logistic regression model, pain, depression, and lower proinflammatory cytokines appear to be the most powerful predictors for fatigue in pSS, identifying fatigue levels with 85% accuracy (19). Considering the role of cytokines in the development of the initial inflammatory response and its inverse association with fatigue, it has been postulated that a potentially maladaptive immune response may contribute to the maintenance of persistent fatigue in a chronic inflammatory state as observed in conditions such as pSS (13, 19, 21).

An interesting theory regarding fatigue in pSS and other infectious and autoimmune inflammatory diseases is the “behavioral response to disease” model. In fact, fatigue is a physiological response and is part of disease behavior, associated with lethargy, anhedonia, apathy, reduced activity, social isolation and depression (22). Findings in animal and clinical studies indicates an activation of the innate immune system in the fatigue mechanisms. It has been demonstrated that sickness behavior is signaled through interleukin-1 (IL-1) receptors on neurons in the brain (22, 23). In humans, there is an increase in IL-1β activity in chronic and autoimmune inflammatory conditions. IL-1β is the final pathway for signaling fatigue in the brain and modulates influx of K+ and Ca++. Peripherally produced IL-1β passes through the blood-brain barrier and reaches neuronal cells in the brain and is also produced by microglia intrathecally. Increased activation of the IL-1 system, as detected by elevated interleukin-1 receptor antagonist (IL-1Ra) levels in cerebrospinal fluid (CSF), was associated with more fatigue in pSS (23). IL-1β has also been implicated in depression and could explain why fatigue and depression are so closely associated (24).

In a comparison of fatigued (n=22) and non-fatigued (n=23) pSS patients 16 serum proteins were differentially expressed (25). By multiplex proteomics it was possible to distinguish fatigued from non-fatigued pSS patients and to identify a “fatigue-signature”. Interestingly, the proteins were involved in inflammatory mechanisms, with neurological and metabolic functions. Top up-regulated proteins included neuroactive synaptosomal-associated protein 25 (SNAP-25), alpha-enolase (ENO1) and ubiquitin carboxyl-terminal hydrolase isozyme L1 (UCHL1). Furthermore, the proinflammatory mediator IL-36α and several complement factors were up-regulated in fatigued compared to non-fatigued patients with pSS (25). Aberrancies in IL-36α are described in several neurological, cognitive and psychological disorders (13).

Proteomic analysis of cerebrospinal fluid in 20 patients with pSS revealed 15 top discriminatory proteins among patients with high and low fatigue. Among these were apolipoprotein-A4, hemopexin, pigment epithelium-derived factor, secretogranin-1, secretogranin-3, selenium-binding protein-1, and complement factor B, with important roles in regulation of innate immunity, cellular stress defense, and/or functions in the central nervous system, and some downregulate inflammation. An important sleep regulator, Hypocretin-1, was also increased in pSS and can influence fatigue by an IL-1β independent mechanism (26).

The role of type II interferons (IFN-γ) in pSS is emerging in a subset of patients characterized by widespread pain, fatigue, and depression (27). Interferon-γ is a major inducer of indoleamine 2,3 dioxygenase (IDO) in many cell types, including fibroblasts, endothelial cells, tumor cells, monocyte-derived macrophages, mesenchymal stromal cells, and dendritic cells. Tryptophan degradation by IDO1 yields a series of catabolites collectively known as kynurenines. The main effect of kynurenines on immune system is inducing TReg response and reducing hyperinflammatory response. It has been postulated that activation of IDO1 may be responsible for manifestations such as hyperalgesia, pain, and depression. This is supported by the evidence that IDO1 pathway activation, measured by the increased Kynurinine/tryptophan ratio in peripheral blood, has been observed in other conditions characterized by chronic pain, and could be an interesting pathway to explain fatigue in pSS (28, 29). Taken together, fatigue signaling pathways appear to be more associated with cell protection and defense than associated with pro-inflammatory pathways and cytokines.

## Treatment of Fatigue

### Pharmacological Treatment

Currently there are no established pharmacological treatment options for idiopathic fatigue. An empiric trial of antidepressant therapy for patients with fatigue and symptoms of depression can be initiated even if the patient does not meet diagnostic criteria for major depression (www.wolterskluver.com/en/solutions/uptodate).

Fatigue can be associated with chronic inflammatory disorders such as RA and pSS, conditions often treated with anti-inflammatory drugs. However, if an underlying condition or inflammation is suspected to cause or contribute to the fatigue, the main focus should be on treating the primary disease (30).

The antimalarial drug hydroxychloroquine (HCQ) is the most frequently prescribed disease modifying antirheumatic drug (DMARD) for patients with pSS and extraglandular manifestations such as arthralgia, arthritis, myalgia, or fatigue. The efficacy for cardinal symptoms of pSS (dryness, pain, and fatigue) were investigated in 120 patients in a randomized, double-blind parallel-group placebo-controlled trial (the JOQUER trial). In comparison to placebo, the study found limited efficacy of HCQ to improve fatigue (31). A more recent meta-analysis, including randomized controlled trials (RCTs), retrospective studies, and prospective studies using HCQ for treatment of pSS. From four studies and a total of 215 patients (herein the JOQUER trial), the meta-analysis found lower efficacy of HCQ for treatment of fatigue than placebo (32).

In a recent phase II double-blind placebo-controlled clinical trial pSS patients were randomized to treatment with RSLV-132 (n=20) or placebo (n=8) (33). RSLV-132 is a biologic drug containing active human RNase with an increased serum half-life compared to wild-type human RNase. There are many non-coding RNAs in human circulation that have inflammatory gene regulatory functions. It was hypothesized that a reduction of these circulating RNAs in patients with pSS might have a positive effect on symptoms such as fatigue and indeed, the study showed improvement of severe fatigue by four independent patient-reported measures (33).

Fatigue associated to inflammatory conditions bear similarities to IL-1 mediated sickness behavior in animals. In a double-blind randomized placebo-controlled clinical trial the IL-1 receptor antagonist (Anakinra) or placebo was administered to 26 pSS patients with fatigue (21). Fatigue was evaluated by VAS and fatigue severity scale (FSS), but no significant difference in fatigue scores was detected between the groups compared to baseline. However, six out of 12 patients on Anakinra versus one out of 13 patients on the placebo reported a 50% reduction in fatigue VAS.

The CD20 antibody Rituximab is an established treatment for several autoimmune conditions. A randomized, double-blind, placebo-controlled pilot study showed a significant improvement in fatigue VAS in pSS patients (n=17) treated with Rituximab compared to baseline. In addition, a significant reduction in SF-36 was observed (34). However, a later randomized, placebo-controlled, triple-blinded, parallel-group trial including 120 patients with recent-onset pSS and biologically active or systemic pSS could not document significant differences between groups when comparing the primary end points (VAS global disease, pain, fatigue, and dryness). An improvement in VAS fatigue was observed in patients treated with Rituximab, but it did not alleviate symptoms or disease activity (35).

Targeting B cell activating factor (BAFF) with Belimumab was investigated in a bi-centric prospective 1-year open-label trial. Patients (n=30) fulfilled American-European Consensus Group (AECG) classification criteria for pSS (36), were anti-Ro/SSA-positive and had current systemic complications or salivary gland enlargement, early disease (<5 years), or biomarkers of B cell activation. The primary end-point was improvement in two of five items (dryness, fatigue, pain, systemic activity assessed by a physician and/or B cell activation biomarker values). The primary end-point was achieved in 18 (60%), among these were seven patients with a reduction in VAS fatigue (23%). The mean fatigue VAS was non-significantly reduced (37).

The effect of dehydroepiandrosterone (DHEA) (200 mg/day) on improvement of fatigue, well-being and functioning was investigated in women with pSS (n=60) in a double-blind, randomized placebo-controlled clinical trial. Primary outcome measures were general fatigue, depressive mood, mental well-being, and physical functioning. Interestingly, patients from both the DHEA- and placebo-treated group improved significantly on general fatigue, mental well-being, and depressive mood, but not physical functioning. The belief to have used DHEA was a stronger predictor for improvement of fatigue and well-being than the actual use of DHEA. On the other hand, the findings suggest possibilities for cognitive behavioral interventions (38). In a later study, patients with pSS (n=107) and severe fatigue and low levels of serum dehydroepiandrosterone/dehydroepiandrosterone sulfate (DHEA/DHEAS) were given DHEA substitution (50 mg/day) in a multicenter, investigator-based, powered, randomized controlled clinical trial (crossover, washout design) with fatigue as the primary outcome measure. All the MFI-20 subscales and the fatigue VAS improved from baseline, but with negligible differences between the two treatments. Similar to earlier results using pharmacologic doses, substitution treatment in DHEA-deficient and severely fatigued patients with pSS was not better than placebo (39).

### Non-Pharmacological Treatment

The promising results of non-pharmacological studies represent a great potential in the management of fatigue in general. So far, exercise and neuromodulation techniques have shown positive effects on fatigue in pSS.

The association between fatigue and reduced levels of physical activity and cardiorespiratory fitness in pSS (6, 40) led to the hypothesis that physical exercise could reduce fatigue. In a recent randomized intention-to-treat study comprising a supervised walking program, FACIT-fatigue in women with pSS was improved compared to the control group. The walking program also increased cardiorespiratory fitness, exercise tolerance and patient perception of improvement, without exacerbating disease activity. In addition, better fatigue scores were associated with reduced depression and improvements in the physical and mental components of QoL (41). Nordic walking exercise also improved fatigue (VAS) in a previous non-randomized study (42). A supervised resistance exercise program in a randomized controlled trial, although there was no direct assessment of fatigue, improved functional capacity and QoL in women with pSS (43).

Based on the growing evidence that the immune-mediated fatigue mechanism may involve or be modulated by the cholinergic anti-inflammatory reflex by stimulating the vagus nerve, a non-invasive vagus nerve stimulation device (nVNS) was investigated in 15 female pSS patients. The nVNS resulted in a significant decrease in daytime sleepiness and fatigue (Prof-F), reduced levels of TNF-α and IL-1β, and reduced TNF-α production by stimulated whole blood cells (44).

Fatigue in pSS could result from hypoactivity of the hypothalamic-pituitary-adrenal axis (HPA) as a possible consequence of autoantibody-mediated destruction of the adrenal glands. Based on this hypothesis, and the evidence that increased cortical excitability by anodal cortex stimulation is capable of modulating the HPA axis activity, a Brazilian double-blind pilot study randomized 36 female pSS patients to receive transcranial direct-current stimulation (tDCS). The tDCS improved fatigue (ESSPRI and FSS) and seemed to promote greater improvements in mental (ProF-M) than somatic fatigue (ProF-S) (45).

Alternative methods such as acupuncture are also being investigated to alleviate dryness, pain and fatigue; key symptoms of pSS. The protocol for the first randomized and controlled pilot trial of acupuncture on alleviating the symptoms of pSS with relatively long-term follow-up was published in 2017 (46). The result of this, and another ongoing study (**Table 2**) might offer a new option to treat pSS and supply clinical proof that acupuncture has beneficial effects on pSS.

**Table 2:** Clinical studies addressing fatigue in Sjögren’s syndrome as their primary outcome www.clinicaltrials.gov.

NCT #	Study name	Treatment/intervention	Sponsor	N	Inclusion criteria	Primary outcome	Estimated completion
*Biological treatments*		
NCT03100942	A Randomized, Phase 2, Double-blind, Placebo-controlled Study to Assess the Safety and Efficacy of Filgotinib, GS-9876 and GS-4059 in Adult Subjects With Active Sjogren’s Syndrome	Drugs: Lanraplenib (GS-9876; SYK inhibitor), Filgotinib (GS-6034; selektive JAK1 inhibitor), Tirabrutinib (GS-4059; Bruton’s tyrosine kinase inhibitor)	Gilead Sciences Galapagos NV Ono Pharmaceutical Co. Ltd	152	pSS or sSS (36) ESSDAI ≥ 5 Seropositivity for anti-SSA or anti-SSB	VAS global disease, pain, oral dryness, ocular dryness and fatigue	January 10, 2019
NCT04093531	Pilot Trial of Ustekinumab for Primary Sjögren’s Syndrome	Drug: Ustekinumab (monoclonal antibody targeting IL-12 and IL-23)	University of Rochester	15	pSS (47, 48) (score >4)	ESSPRI SF-36 in secondary outcome	December 2021
NCT04129164	A Phase 2 Randomized, Double-blind, Placebo-controlled, Proof of Concept Study to Evaluate the Efficacy and Safety of VIB4920 in Subjects with Sjögren’s Syndrome (SS)	Drug: VIB4920 (fusion protein designed to bind CD40 ligand on activated T cells)	Viela Bio	174	SS (47, 48)	ESSPRI (FACIT-F in secondary outcomes)	April 1, 2022
*Alternative treatments*	
NCT04056221	Effects of Acupuncture on Xerostomia and Xerophthalmia in Sjögren’s Syndrome: A Randomized, Double-blinded Clinical Trial	Acupuncture inserted on the selected acupoints (R6, E6, E2, Ig4, VC24, TA23, B2)	Ana Carolina Fragoso Motta, DDS, PhD	50	SS (47, 48)	ESSPRI	December 2020
NCT03983408	Impact of Korean Red Ginseng on Fatigue in Patients with Rheumatic Disease	Dietary Supplement: Korean Red Ginseng (Chinese herbal supplement)	Hanyang University The Korean Society of Ginseng	120	Sjögren’s syndrome Fatigue for > 3 months	FACIT-F	August 31, 2020
NCT04653935	Fully-remote Trial of a Self-management App for Those Living With Sjögren’s Syndrome: Randomised Pilot and Feasibility Study	Behavioral: Self-management app with intervention components; cognitive- and behavioral self-management techniques (Sjogo app)	Northumbria University Newcastle University Teesside University Versus Arthritis	996	Diagnosed with pSS or sSS (self-reported)	MFIS-5 and ESSPRI	July 2021

A focus group study on patients with established pSS indicated a range of sleep disturbances, and an overlap between the participants sleep and fatigue symptoms, although the fatigue they experienced was very different from “tiredness”. The study participants already utilized a number of strategies to manage their sleep. Cognitive behavioral therapy was viewed as an acceptable intervention, given a rationale for its use and it is tailored for pSS (49). A multidisciplinary approach according to the needs of the patient, including occupational therapy, physiotherapy and/or health psychology improved fatigue VAS scores and were maintained at 6-12 months follow-up in 50 pSS patients (50).

## Discussion

Fatigue is a characteristic symptom of pSS, occurring in about two thirds of the patients. Fatigue (and pain) is an important predictor of poor QoL regardless of age, schooling, marital status, work disability, fibromyalgia and disease activity (11). Fatigue in pSS is multifactorial and is clinically related to sleep and mood disorders (20). On the other hand, it is not associated with systemic disease activity (11, 17) and has an inverse correlation with pro-inflammatory cytokines (18, 19).

The biological mechanisms involved in pSS fatigue are still not fully understood. Recent studies using proteomic analysis have shown the expression of proteins in the blood (SNAP-25, ENO1, UCHL1, IL-36a and complement factors) and in the CSF (apolipoprotein-A4, hemopexin, pigment epithelium-derived factor, secretogranin-1, secretogranin-3, selenium-binding protein-1, and complement factor B) in fatigued compared to non-fatigued pSS (13, 26). With important roles in regulation of innate immunity, cellular stress defense, and/or functions in the central nervous system, supporting the hypothesis that fatigue signaling pathways appear to be more associated with cell protection and defense than with pro-inflammatory pathways and cytokines. Future studies should try to confirm this hypothesis and explore the role of type II (IFN-γ) and Kynurinines pathway (27–29). In this review we focus on biomarkers of the immune system involved in fatigue in pSS. Fatigue is multidimensional and associated with neurocognitive, neuroendocrine, environmental and behavioral components (51). Additionally, many patients with pSS have poor sleep, and correlated with fatigue (52). Sleep quality and cognitive compromises being associated with fatigue, future studies could address whether such factors could be useful as surrogate markers for fatigue in pSS.

Hydroxychloroquine, DHEA, and rituximab failed to improve fatigue in controlled studies (35, 37, 38). The RSLV-132, an RNase Fc fusion protein, in a phase II study showed improvement of severe fatigue as determined by four independent patient-reported measures of fatigue (33). Ongoing clinical trials are testing new targets on fatigue in pSS, such as, Lanraplenib (GS-9876), Filgotinib (GS-6034), Tirabrutinib (GS-4059), CDZ173, and VIB4920 (**Table 2**).

Exercise and neuromodulation techniques have shown positive effects on fatigue in pSS (41, 43, 45). Non-pharmacological studies represent a great potential in the management of fatigue in general. High-quality RCTs for potential non-pharmacological interventions such as different types of aerobic exercise, resistance exercise, nVNS, and tDCS must be performed, as well as studies with long-term follow-up.

Different non-pharmacological interventions for fatigue have been investigated in other rheumatic diseases (53), and self-management training through patient education programs, cognitive-behavioral therapy, and mindfulness could also be tested in pSS. The positive effect of placebo observed in therapeutic clinical studies (38, 39) does indeed give promise for cognitive behavioral interventions. Ongoing studies investigating treatment of SS with fatigue as the primary outcomes are listed in **Table 2** and include biological agents as well as alternative treatments such as dietary supplements and acupuncture. In the future, artificial intelligence and digital tools may also become helpful to generate more objective clinical endpoints.

## Author Contributions

EM: idea and concept of manuscript, writing of the manuscript. SM: concept of manuscript, writing and revision of the manuscript. DH: idea and concept of manuscript, writing of the manuscript. VV: initiative and concept of manuscript, lay-out, writing and revision of the manuscript. MJ: initiative and concept of manuscript, lay-out, draft, revision and submission of the manuscript. All authors contributed to the article and approved the submitted version.

## Funding

The study was funded by the University of Bergen, the University Hospital of Federal University of Espírito Santo, and the Oral Health Centre of Expertise in Western Norway.

## Conflict of Interest

The authors declare that the research was conducted in the absence of any commercial or financial relationships that could be construed as a potential conflict of interest.

## References

[1] Qin B Wang J Yang Z Yang M Ma N Huang F. Epidemiology of Primary Sjögren’s Syndrome: A Systematic Review and Meta-Analysis. Ann Rheum Dis (2015) 74(11):1983–9. 10.1136/annrheumdis-2014-205375 24938285

[2] Jonsson R Haga HJ Gordon TP. Sjögren’s Syndrome. In: Koopman WJ, editor. Arthritis and Allied Conditions - A Textbook of Rheumatology, 14th ed, vol. p . Philadelphia: Lippincott Williams & Wilkins (2001). p. 1736–59.

[3] Mengshoel AM Norheim KB Omdal R. Primary Sjögren’s Syndrome: Fatigue is an Ever-Present, Fluctuating, and Uncontrollable Lack of Energy. Arthritis Care Res (2014) 66(8):1227–32. 10.1002/acr.22263 24339344

[4] Retamozo S Brito-Zeron P Ramos-Casals M. Prognostic Markers of Lymphoma Development in Primary Sjögren Syndrome. Lupus (2019) 28(8):923–36. 10.1177/0961203319857132 31215845

[5] Ng WF Bowman SJ. Primary Sjögren’s Syndrome: Too Dry and Too Tired. Rheumatol (Oxford) (2010) 49(5):844–53. 10.1093/rheumatology/keq009 20147445

[6] Strömbeck B Ekdahl C Manthorpe R Jacobsson LT. Physical Capacity in Women With Primary Sjögren’s Syndrome: A Controlled Study. Arthritis Rheum (2003) 49(5):681–8. 10.1002/art.11384 14558054

[7] Cornec D Devauchelle-Pensec V Mariette X Jousse-Joulin S Berthelot JM Perdriger A. Severe Health-Related Quality of Life Impairment in Active Primary Sjögren’s Syndrome and Patient-Reported Outcomes: Data From a Large Therapeutic Trial. Arthritis Care Res (2017) 69(4):528–35. 10.1002/acr.22974 27390310

[8] Haldorsen K Bjelland I Bolstad AI Jonsson R Brun JG. A Five-Year Prospective Study of Fatigue in Primary Sjögren’s Syndrome. Arthritis Res Ther (2011) 13(5):R167. 10.1186/ar3487 21996338 PMC3308101

[9] Hackett KL Newton JL Frith J Elliott C Lendrem D Foggo H. Impaired Functional Status in Primary Sjögren’s Syndrome. Arthritis Care Res (2012) 64(11):1760–4. 10.1002/acr.21738 23111856

[10] Cui Y Li L Xia L Zhao Q Chen S Fu T. The Impact of Disease Activity and Psychological Status on Quality of Life for Chinese Patients With Primary Sjögren’s Syndrome. Patient Prefer Adherence (2018) 12:1513–9. 10.2147/PPA.S163417 PMC6110269 30174416

[11] Dias LH Miyamoto ST Giovelli RA Magalhães CIM Valim V. Pain and Fatigue are Predictors of Quality of Life in Primary Sjögren’s Syndrome. Adv Rheumatol (2021) 61(1):28. 10.1186/s42358-021-00181-9 34051867

[12] Gairy K Ruark K Sinclair SM Brandwood H Nelsen L. An Innovative Online Qualitative Study to Explore the Symptom Experience of Patients With Primary Sjögren’s Syndrome. Rheumatol Ther (2020) 7(3):601–15. 10.1007/s40744-020-00220-9 PMC7410917 32725407

[13] Omdal R Mellgren SI Norheim KB. Pain and Fatigue in Primary Sjögren’s Syndrome. Rheumatol (Oxford) (2019) kez027. 10.1093/rheumatology/kez027 30815693

[14] Theander L Strombeck B Mandl T Theander E. Sleepiness or Fatigue? Can We Detect Treatable Causes of Tiredness in Primary Sjögren’s Syndrome? Rheumatol (Oxford) (2010) 49(6):1177–83. 10.1093/rheumatology/keq023 20308122

[15] Hewlett S Dures E Almeida C. Measures of Fatigue: Bristol Rheumatoid Arthritis Fatigue Multi-Dimensional Questionnaire (Braf MDQ), Bristol Rheumatoid Arthritis Fatigue Numerical Rating Scales (Braf NRS) for Severity, Effect, and Coping, Chalder Fatigue Questionnaire (Cfq), Checklist Individual Strength (CIS20R and CIS8R), Fatigue Severity Scale (Fss), Functional Assessment Chronic Illness Therapy (Fatigue) (Facit-F), Multi-Dimensional Assessment of Fatigue (Maf), Multi-Dimensional Fatigue Inventory (Mfi), Pediatric Quality Of Life (Pedsql) Multi-Dimensional Fatigue Scale, Profile of Fatigue (Prof), Short Form 36 Vitality Subscale (Sf-36 VT), and Visual Analog Scales (Vas). Arthritis Care Res (2011) 63 Suppl 11:S263–86. 10.1002/acr.20579 22588750

[16] Miyamoto ST Lendrem DW Ng WF Hackett KL Valim V. Managing Fatigue in Patients With Primary Sjögren’s Syndrome: Challenges and Solutions. Open Access Rheumatol (2019) 11:77–88. 10.2147/OARRR.S167990 31118841 PMC6503647

[17] Lendrem D Mitchell S McMeekin P Gompels L Hackett K Bowman S. Do the EULAR Sjögren’s Syndrome Outcome Measures Correlate With Health Status in Primary Sjögren’s Syndrome? Rheumatol (Oxford) (2015) 54(4):655–9. 10.1093/rheumatology/keu361 25240612

[18] Howard Tripp N Tarn J Natasari A Gillespie C Mitchell S Hackett KL. Fatigue in Primary Sjögren’s Syndrome is Associated With Lower Levels of Proinflammatory Cytokines. RMD Open (2016) 2(2):e000282. 10.1136/rmdopen-2016-000282 27493792 PMC4964201

[19] Davies K Mirza K Tarn J Howard-Tripp N Bowman SJ Lendrem D. Fatigue in Primary Sjögren’s Syndrome (pSS) is Associated With Lower Levels of Proinflammatory Cytokines: A Validation Study. Rheumatol Int (2019) 39(11):1867–73. 10.1007/s00296-019-04354-0 PMC6791914 31250166

[20] Hackett KL Davies K Tarn J Bragg R Hargreaves B Miyamoto S. Pain and Depression are Associated With Both Physical and Mental Fatigue Independently of Comorbidities and Medications in Primary Sjögren’s Syndrome. RMD Open (2019) 5(1):e000885. 10.1136/rmdopen-2018-000885 31168409 PMC6525628

[21] Norheim KB Harboe E Göransson LG Omdal R. Interleukin-1 Inhibition and Fatigue in Primary Sjögren’s Syndrome–A Double Blind, Randomised Clinical Trial. PloS One (2012) 7(1):e30123. 10.1371/journal.pone.0030123 22253903 PMC3254637

[22] Dantzer R O’Connor JC Freund GG Johnson RW Kelley KW. From Inflammation to Sickness and Depression: When the Immune System Subjugates the Brain. Nat Rev Neurosci (2008) 9(1):46–56. 10.1038/nrn2297 18073775 PMC2919277

[23] Harboe E Tjensvoll AB Vefring HK Göransson LG Kvaloy JT Omdal R. Fatigue in Primary Sjögren’s Syndrome–A Link to Sickness Behaviour in Animals? Brain Behav Immun (2009) 23(8):1104–8. 10.1016/j.bbi.2009.06.151 19560535

[24] Kim JM Kang HJ Kim JW Bae KY Kim SW Kim JT. Associations of Tumor Necrosis Factor-alpha and Interleukin-1beta Levels and Polymorphisms With Post-Stroke Depression. Am J Geriatr Psychiatry (2017) 25(12):1300–8. 10.1016/j.jagp.2017.07.012 28844626

[25] Bodewes ILA van der Spek PJ Leon LG Wijkhuijs AJM van Helden-Meeuwsen CG Tas L. Fatigue in Sjögren’s Syndrome: A Search for Biomarkers and Treatment Targets. Front Immunol (2019) 10:312. 10.3389/fimmu.2019.00312 30863411 PMC6399420

[26] Larssen E Brede C Hjelle A Tjensvoll AB Norheim KB Bårdsen K. Fatigue in Primary Sjögren’s Syndrome: A Proteomic Pilot Study of Cerebrospinal Fluid. SAGE Open Med (2019) 7:2050312119850390. 10.1177/2050312119850390 31205695 PMC6537061

[27] Del Papa N Minniti A Lorini M Carbonelli V Maglione W Pignataro F. The Role of Interferons in the Pathogenesis of Sjögren’s Syndrome and Future Therapeutic Perspectives. Biomolecules (2021) 11(2):251. 10.3390/biom11020251 33572487 PMC7916411

[28] de Oliveira FR Fantucci MZ Adriano L Valim V Cunha TM Louzada-Junior P. Neurological and Inflammatory Manifestations in Sjögren’s Syndrome: The Role of the Kynurenine Metabolic Pathway. Int J Mol Sci (2018) 19(12):3953. 10.3390/ijms19123953 PMC6321004 30544839

[29] Sardenberg WM Santos MCLFS Skarstein K Carvalho Caser L Brun JG Ulvik A. Acinar Adipose Tissue Infiltration in Salivary Gland Biopsy is Associated With kynurenines-Interferon-gamma Pathway Inflammation Biomarkers. Clin Exp Rheumatol (2020) 38 Suppl 126(4):27–33. 33095140

[30] Marin H Menza MA. Specific Treatment of Residual Fatigue in Depressed Patients. Psychiatry (Edgmont) (2004) 1(2):12–8. PMC3012615 21197374

[31] Gottenberg JE Ravaud P Puechal X Le Guern V Sibilia J Goeb V. Effects of Hydroxychloroquine on Symptomatic Improvement in Primary Sjögren Syndrome: The JOQUER Randomized Clinical Trial. JAMA (2014) 312(3):249–58. 10.1001/jama.2014.7682 25027140

[32] Wang SQ Zhang LW Wei P Hua H. Is Hydroxychloroquine Effective in Treating Primary Sjögren’s Syndrome: A Systematic Review and Meta-Analysis. BMC Musculoskelet Disord (2017) 18(1):186. 10.1186/s12891-017-1543-z 28499370 PMC5427554

[33] Posada J Valadkhan S Burge D Davies K Tarn J Casement J. Improvement of Severe Fatigue Following Nuclease Therapy in Patients With Primary Sjögren’s Syndrome: A Randomized Clinical Trial. Arthritis Rheumatol (2021) 73(1):143–50. 10.1002/art.41489 PMC7839752 32798283

[34] Dass S Bowman SJ Vital EM Ikeda K Pease CT Hamburger J. Reduction of Fatigue in Sjögren Syndrome With Rituximab: Results of a Randomised, Double-Blind, Placebo-Controlled Pilot Study. Ann rheumatic Dis (2008) 67(11):1541–4. 10.1136/ard.2007.083865 18276741

[35] Devauchelle-Pensec V Mariette X Jousse-Joulin S Berthelot JM Perdriger A Puechal X. Treatment of Primary Sjögren Syndrome With Rituximab: A Randomized Trial. Ann Internal Med (2014) 160(4):233–42. 10.7326/M13-1085 24727841

[36] Vitali C Bombardieri S Jonsson R Moutsopoulos HM Alexander EL Carsons SE. Classification Criteria for Sjögren’s Syndrome: A Revised Version of the European Criteria Proposed by the American-European Consensus Group. Ann rheumatic Dis (2002) 61(6):554–8. 10.1136/ard.61.6.554 PMC1754137 12006334

[37] Mariette X Seror R Quartuccio L Baron G Salvin S Fabris M. Efficacy and Safety of Belimumab in Primary Sjögren’s Syndrome: Results of the BELISS Open-Label Phase II Study. Ann rheumatic Dis (2015) 74(3):526–31. 10.1136/annrheumdis-2013-203991 24347569

[38] Hartkamp A Geenen R Godaert GL Bootsma H Kruize AA Bijlsma JW. Effect of Dehydroepiandrosterone Administration on Fatigue, Well-Being, and Functioning in Women With Primary Sjögren Syndrome: A Randomised Controlled Trial. Ann rheumatic Dis (2008) 67(1):91–7. 10.1136/ard.2007.071563 17545193

[39] Virkki LM Porola P Forsblad-d’Elia H Valtysdottir S Solovieva SA Konttinen YT. Dehydroepiandrosterone (DHEA) Substitution Treatment for Severe Fatigue in DHEA-Deficient Patients With Primary Sjögren’s Syndrome. Arthritis Care Res (2010) 62(1):118–24. 10.1002/acr.20022 20191499

[40] Ng WF Miller A Bowman SJ Price EJ Kitas GD Pease CT. Physical Activity But Not Sedentary Activity Is Reduced in Primary Sjögren’s Syndrome. Rheumatol Int (2017) 37(4):623–31. 10.1007/s00296-016-3637-6 PMC5357288 28013357

[41] Miyamoto ST Valim V Carletti L Ng WF Perez AJ Lendrem DW. Supervised Walking Improves Cardiorespiratory Fitness, Exercise Tolerance, and Fatigue in Women With Primary Sjögren’s Syndrome: A Randomized-Controlled Trial. Rheumatol Int (2019) 39(2):227–38. 10.1007/s00296-018-4213-z 30604204

[42] Strömbeck BE Theander E Jacobsson LT. Effects of Exercise on Aerobic Capacity and Fatigue in Women With Primary Sjögren’s Syndrome. Rheumatol (Oxford) (2007) 46(5):868–71. 10.1093/rheumatology/kem004 17308315

[43] Minali PA Pimentel C de Mello MT Lima G Dardin LP Garcia A. Effectiveness of Resistance Exercise in Functional Fitness in Women With Primary Sjögren’s Syndrome: Randomized Clinical Trial. Scandinavian J Rheumatol (2020) 49(1):47–56. 10.1080/03009742.2019.1602880 31244376

[44] Tarn J Legg S Mitchell S Simon B Ng WF. The Effects of Noninvasive Vagus Nerve Stimulation on Fatigue and Immune Responses in Patients With Primary Sjögren’s Syndrome. Neuromodulation (2019) 22(5):580–5. 10.1111/ner.12879 30328647

[45] Pinto ACPN Piva SR Vieira AGDS Gomes SGCN Rocha AP Tavares DRB. Transcranial Direct Current Stimulation for Fatigue in Patients With Sjögren’s Syndrome: A Randomized, Double-Blind Pilot Study. Brain Stimul (2021) 14(1):141–51. 10.1016/j.brs.2020.12.004 33340767

[46] Jiang Q Zhang H Pang R Chen J Liu Z Zhou X. Acupuncture for Primary Sjögren Syndrome (pSS) on Symptomatic Improvements: Study Protocol for a Randomized Controlled Trial. BMC Complement Altern Med (2017) 17(1):61. 10.1186/s12906-017-1559-9 28103850 PMC5248458

[47] Shiboski CH Shiboski SC Seror R Criswell LA Labetoulle M Lietman TM. 2016 American College of Rheumatology/European League Against Rheumatism Classification Criteria for Primary Sjögren’s Syndrome: A Consensus and Data-Driven Methodology Involving Three International Patient Cohorts. Ann Rheum Dis (2017) 76(1):9–16. 10.1136/annrheumdis-2016-210571 27789466

[48] Shiboski CH Shiboski SC Seror R Criswell LA Labetoulle M Lietman TM. 2016 American College of Rheumatology/European League Against Rheumatism Classification Criteria for Primary Sjögren’s Syndrome: A Consensus and Data-Driven Methodology Involving Three International Patient Cohorts. Arthritis Rheumatol (2017) 69(1):35–45. 10.1136/annrheumdis-2016-210571 27785888 PMC5650478

[49] Hackett KL Deary V Deane KH Newton JL Ng WF Rapley T. Experience of Sleep Disruption in Primary Sjögren’s Syndrome: A Focus Group Study. Br J Occup Ther (2018) 81(4):218–26. 10.1177/0308022617745006 PMC5881782 29657352

[50] Hackett KL Deane KHO Newton JL Deary V Bowman SJ Rapley T. Mixed-Methods Study Identifying Key Intervention Targets to Improve Participation in Daily Living Activities in Primary Sjögren’s Syndrome Patients. Arthritis Care Res (2018) 70(7):1064–73. 10.1002/acr.23536 PMC6033158 29409110

[51] Mavragani CP Fragoulis GE Moutsopoulos HM. Endocrine Alterations in Primary Sjögren’s Syndrome: An Overview. J Autoimmun (2012) 39(4):354–8. 10.1016/j.jaut.2012.05.011 22695186

[52] Dardin LP Garcia ABA Gazoni FM Santos FCD Mello MT Trevisani VFM. Correlation of Sleep Quality With Fatigue and Disease Activity Among Patients With Primary Sjögren’s Syndrome: A Cross-Sectional Study. Sao Paulo Med J (2020) 138(2):146–51. 10.1590/1516-3180.2019.0251.r1.1912019 PMC9662840 32159602

[53] Cramp F Hewlett S Almeida C Kirwan JR Choy EH Chalder T. Non-Pharmacological Interventions for Fatigue in Rheumatoid Arthritis. Cochrane Database Syst Rev (2013) (8):CD008322. 10.1002/14651858.CD008322.pub2 PMC11748118 23975674

